# Redundant trials can be prevented, if the EU clinical trial regulation is applied duly

**DOI:** 10.1186/s12910-020-00536-9

**Published:** 2020-10-28

**Authors:** Daria Kim, Joerg Hasford

**Affiliations:** 1 Research Fellow, Max Planck Institute for Innovation and Competition, Marstallplatz 1, 81545 Munich, Germany; 2 Ludwig-Maximilians-University of Munich, The Institute for Medical Information Processing, Biometry, and Epidemiology, and Chairman of the Permanent Working Party of Research Ethics Committees in Germany, Scharnitzerstaße 7, 82166 Gräfelfing, Germany

**Keywords:** Clinical trials, EU clinical trials regulation, Research ethics, Research ethics committees, Research redundancy, Systematic review, Trial authorisation, Trial methodology

## Abstract

The problem of wasteful clinical trials has been debated relentlessly in the medical community. To a significant extent, it is attributed to redundant trials – studies that are carried out to address questions, which can be answered satisfactorily on the basis of existing knowledge and accessible evidence from prior research. This article presents the first evaluation of the potential of the EU Clinical Trials Regulation 536/2014, which entered into force in 2014 but is expected to become applicable at the end of 2021, to prevent such trials. Having reviewed provisions related to the trial authorisation, we propose how certain regulatory requirements for the assessment of trial applications can and should be interpreted and applied by national research ethics committees and other relevant authorities in order to avoid redundant trials and, most importantly, preclude the unnecessary recruitment of trial participants and their unjustified exposure to health risks.

## Keypoints

*What is known?*

- The problem of wasteful clinical trials has been exposed by empirical studies and debated intensely within the medical community.
- To a significant extent, wasteful research can be attributed to redundant trials – ie trials that intend to address questions that can be answered satisfactorily on the basis of evidence gathered in earlier studies.

*What does this article add?*

- Certain provisions under the EU Clinical Trials Regulation, which is expected to become applicable in 2021/2022, can and should be interpreted and applied in a way that can empower institutions responsible for trial authorisation – research ethics committees (RECs) and national competent authorities (NCAs) – to play a more prominent role in preventing redundant trials.

*What is proposed?*

Applicants for the trial authorisation shall

- justify a newly proposed trial by demonstrating that it addresses an *outstanding clinical uncertainty* in light of the available evidence relevant for the research question and the outcome of interest at issue; and
- show how the synthesis of earlier research *informed the design* of a proposed trial.

- Where no systematic review exists, applicants should make their best effort to identify and synthesise knowledge gained in prior studies.
- Research Ethics Committees and drug regulatory authorities need to be properly staffed to effectively reduce redundant interventional studies.

## Background

The problem of wasteful – ie unregistered [1, 2] biased [3], unreported [4, 5], unpublished [6, 7], clinically irrelevant, inadequately designed, or otherwise wasteful [8–19] – trials has been debated relentlessly in the medical research community. In 2009, Iain Chalmers and Paul Glasziou made a staggering claim that up to 85% of clinical trials can be cumulatively considered to be wasteful ([19], p. 88). By ‘waste’, the authors broadly refer to deficiencies in the ways randomised trials are designed, conducted, analysed, reported, regulated, and managed.

In general terms, a trial can be deemed to be wasteful if it does not produce new robust medical knowledge that can justify health risks borne by study participants, research efforts of investigators, and the allocated financial and other resources. Earlier research has analysed the causes and scope of the problem. The critical question is what can be done in order to eliminate or, at least, alleviate it. While opinions differ as to who – regulators [11], investigators [16], funders [16], health care professionals [20], ethics committees and journals [21], methodologists and medical statisticians [22] – should take the lead, it is clear that a unified and systematic approach needs to be implemented at all levels of decision-making.

This article focuses on the issue of redundant randomised clinical trials (RCT) – ie trials that do not contribute to the stock of biomedical knowledge relevant for clinical practice in a way that would justify the risks and costs involved. The problem is often attributed to the insufficient consideration of earlier findings. While redundancy can be difficult to detect, it is highly important that such trials are precluded at the stage of the trial application – prior to the enrolment of study participants.

Commentators have long been advocating – albeit with little hope [23, 24] – that greater scrutiny should be exercised with regard to the applications for clinical trials, especially as far as their justification vis-à-vis prior research and relevance for clinical practice are concerned. The purpose of this article is to examine the potential of the EU Regulation 536/2014 [25] (hereinafter the EU Clinical Trials Regulation) to tackle the problem of research redundancy. While the Regulation was adopted and entered into force in 2014, it will become applicable upon the publication of the notice confirming the full functionality of the EU portal and the EU database by the European Commission ([25], Article 99). Currently the full functionality of the EU Portal is expected at end of 2021 or early 2022. In this context, the present analysis is pertinent and highly timely.

In what follows, we describe the problem of redundancy in clinical trials, review the earlier discourse in the medical community and findings of empirical studies on this subject. Upon identifying key provisions under the EU Clinical Trials Regulation that are closely related to the justification of a trial against the background of prior research, we assess whether they can be leveraged to eliminate redundancy. Further, we propose the interpretation that can be instrumental in preventing redundant trials and discuss critical factors of applying our recommendations. We conclude by reinforcing the idea that, while the EU Clinical Trials Regulation strives to promote competitiveness of European clinical research, it is methodological quality and ethical integrity that shall be viewed as the core aspects.

## Main text

### The problem of redundant trials

#### When is a trial considered to be redundant?

A universal definition of a ‘redundant trial’ hardly exists. Redundancy occurs if a trial intends to investigate a question that can be ‘answered satisfactorily with existing evidence’ ([19], p. 87), or where the outcome of interest does not involve genuine, clinically relevant uncertainty. According to the International Ethical Guidelines for Health-related Research Involving Humans, such studies, even if rigorously designed, ‘lack social value’ because the research question at issue has already been ‘successfully addressed in prior research’ [26], p. 2]. Commentators have also referred to redundant trials as ‘unnecessary duplication of research efforts’ ([9], p. 159; [17], p. 4). A clarification is necessary: in a strict sense, a duplicative trial would mean a trial that intends to test an identical medicinal product for the identical condition as an earlier trial. The only time when such duplication can be sanctioned is when two phase III trials are conducted to gather substantial evidence on efficacy and safety, typically requested by drug authorities such as the European Medicines Agency in the European Union and the Food and Drug Administration in the U.S. Beyond this requirement, there is no regulatory need for duplication. The probability of a trial conducted for the purpose of generic drug approval shall also be excluded, since in most, if not all, jurisdictions generic drugs are exempted from conducting full-scale clinical trials in order to be authorised for marketing.

Furthermore, redundant trials should not, by any means, be confused with phase IV studies that intend to further improve and refine the dosage recommendation or the understanding of benefit-risk relationship in general or specific populations, and/or to find less typical adverse reactions of medicinal products approved for marketing. It is important to emphasise that the problem of redundancy is not phase-specific but *case*-specific. Based on the empirical studies on this issue (partially summarised in Table 1), the concept of redundancy ought to be understood broadly in relation to therapeutic subcategories. This, by no means, implies that further studies investigating new interventions in a given therapeutic class or subclass are redundant per se in situations where there is a standard treatment. Quite on the contrary, there can be numerous aspects that might need to be investigated in further studies. The decisive factor is not whether a standard treatment exists or not, but how a newly proposed trial is designed, in particular, the extent to which evidence from prior studies related to the research question and the outcome of interest has been taken into consideration, and whether a subsequent trial intends to address genuine uncertainty and novel aspects of a treatment. Having said that, it is worth noting that, in the case of phase IV trials, the problem in practice might be reverse, ie there is likely to be underproduction of comparative evidence rather than excessive studies on therapeutic use of treatments in the post-marketing authorisation phase.

**Table 1:** An overview of studies measuring the scale of redundancy in RCTs

Study	Objective	Method	Sample	Results	Conclusions
Lau et al. (1992) [27]	To demonstrate that ‘searching and monitoring the clinical literature and performing cumulative meta-analyses can […] supply practitioners and policy makers with up-to-date information on emerging and established [medical] advances’ ([27], p. 248).	Cumulative meta-analyses of clinical trials that evaluated 15 treatments and preventive measures for acute myocardial infarction.	Trials conducted between 1959 and 1988 that investigated the use of intravenous streptokinase as thrombolytic therapy for acute infarction.	A consistent, statistically significant reduction in total mortality was achieved in 1973 upon the completion of eight trials involving 2432 patients; 25 subsequent trials, in which 34,542 patients were enrolled, had little or no effect on the odds ratio establishing efficacy.	Clinical trials are ‘part of a continuum, and those that have gone before must be considered when new ones are planned’ ([27], p. 253).
Fergusson et al. (2005) [21]	To evaluate the impact of systematic reviews of RCTs on the design of subsequent trials.	Cumulative meta-analyses of all RCTs of aprotinin using placebo controls or no active control treatment. Parameters of collected data included the study primary outcomes, objectives, the presence of a systematic review as a part of the background and/or rationale for the study, the number of previously published RCTs cited.	All RCTs of aprotinin conducted between 1987 and 2002 reporting an endpoint of perioperative transfusion.	64 RCTs meeting the selection criteria were identified with the median trial size ranging between 20 and 1784 trial participants. A cumulative meta-analysis showed that aprotinin significantly decreased the need for perioperative transfusion, stabilizing at an odds ratio of 0.25 by the 12th study published in 1992. Thereafter, the upper limit of the confidence interval did not exceed 0.65 and results were similar in all subgroups. Citation of previous RCTs was low – on average, only 20% of relevant prior trials was cited. Only 7 of 44 of subsequent reports referenced the largest trial, which was 28 times larger than the median trial size.	Investigators evaluating aprotinin ‘were not adequately citing previous research, resulting in a large number of RCTs being conducted to address efficacy questions that prior trials had already definitively answered’ ([21], p. 218).
Cooper, Jones, Sutton (2005) [28]	To assess the extent, to which Cochrane systematic reviews are taken into account in the design of new trials.	A survey among authors of published studies added in the updated Cochrane reviews. Authors were asked if they had used the 1996 Cochrane or other reviews in designing their trials.	All studies included in the 2002 and 2003 updates of the 1996 Cochrane review (overall, 33 Cochrane reviews).	Of 32 authors of eligible studies newly included in the updated Cochrane reviews, 24 responded. Eleven respondents were aware of the relevant Cochrane review at the time of designing the study. In eight cases the design of the new study had been influenced by a review; in two this was the relevant Cochrane review.	Cochrane or other systematic reviews are used in the designing of new studies to a rather limited extent ([28], p. 260).
Goudie et al. (2010) [29]	To define the extent to which previous trials were considered in the design of new trials (eg in the calculation of the sample size).	The assessment of a sample of RCTs to establish whether authors considered previous trials when designing own trials.	27 RCTs published in the leading medical journals in 2007.	Only a small fraction of the trials in the analysed sample referenced the relevant meta-analyses and related the results of the trial to previous research.	Previous evidence from trials ‘is not used (or not reported to be used) as extensively as it could be in justifying, designing, and reporting RCTs’ ([29], p. 984).
Robinson and Goodman (2011) [24]	To evaluate the extent, to which the reports of RCTs cite prior trials addressing the same interventions.	Meta-analyses published in 2004 that combined four or more trials were identified; within each meta-analysis, the extent to which each trial report cited the trials that preceded it by more than one year was assessed.	227 meta-analyses comprising 1523 trials across various health care disciplines published from 1963 to 2004.	Less than 25% of the eligible prior RCTs was cited. The percentage of ‘ignored RCTs [was] increasing as the number of those RCTs increased, [while] the proportion of trials citing no prior evidence stayed constant as the evidence accumulated’ ([24], p. 54). The reports that did cite individual trials in the introduction and discussion sections, were not integrating their findings with the cited trials. In several cases, the investigators ‘claimed to be the first trial even when many trials preceded them’ (ibid).	Further research is needed to explore the explanations for and consequences of the under-citation of earlier research. ‘Potential implications [of under-citation] include ethically unjustifiable trials, wasted resources, incorrect conclusions, and unnecessary risks for trial participants’ ([24], p. 50).
Ker et al. (2012) [30]	To assess the effect of tranexamic acid on blood transfusion, thromboembolic events, and mortality in surgical patients.	Systematic review and meta-analysis.	RCTs comparing tranexamic acid with no tranexamic acid or placebo in surgical patients. 129 trials, totalling 10,488 patients, carried out between 1972 and 2011 were included.	‘A statistically significant effect of tranexamic acid on blood transfusion was first observed after publication of the third trial in 1993. Although subsequent trials have increased the precision of the point estimate, no substantive change has occurred in the direction or magnitude of the treatment effect.’ ([30], p. 3)	‘Reliable evidence that tranexamic acid reduces blood transfusion in surgical patients has been available for many years. […] those planning further placebo controlled trials should … focus their efforts on resolving the uncertainties about the effect of tranexamic acid on thromboembolic events and mortality.’ ([30], p. 3)
Jones et al. (2013) [31]	To examine how systematic reviews of earlier trials had been used to inform the design of new RCTs.	Review of RCTs with regard to the following parameters: the justification of treatment comparison, choice of frequency or dose, selection (or definition) of an outcome, recruitment and consent rates, sample size (margin of equivalence or non-inferiority, size of difference, control group event rate, measure of variability and loss to follow up adjustment), length of follow-up, withdrawals, missing data or adverse events.	The documentation related to RCTs funded under the National Institute for Health Research Health Technology Assessment programme in the UK in 2006, 2007 and 2008 and included applications for funding and project descriptions of 48 RCTs.	About half of the examined applications for funding in fact used the cited review in order to inform the trial design, in particular, the selection and definition of the outcomes, the calculation of the sample size and the duration of follow up.	Guidelines for applicants and funders were proposed as to how systematic reviews can be used to optimise the design and planning of new RCTs.
Clarke, Brice, Chalmers (2014) [17]	To provide ‘the most comprehensive collection of cumulative meta-analysis of studies of healthcare interventions’, and to explore that cumulative evidence in the context of unnecessary duplication of research efforts ([17], p. 2).	A systematic review of the findings of cumulative meta-analyses of all studies examining effects of clinical interventions published between 1992 and 2012 and accessible through PubMed, MEDLINE, EMBASE, the Cochrane Methodology Register and Science Citation Index.	50 eligible reports including over 1500 cumulative meta-analyses.	Four cumulative meta-analyses have shown ‘how replications have challenged initially favourable results where the early trials were favourable but not statistically significant’ ([17], p. 3). Two cumulative meta-analyses have shown ‘how replications have sometimes challenged initially unfavourable results’ (ibid). 22 cumulative meta-analyses demonstrated that ‘a systematic review of existing research would have reduced uncertainty about an intervention’ (ibid). Some trials were ‘much too small’ to resolve uncertainties exposed by the cumulative meta-analyses (ibid).	‘… had researchers assessed systematically what was already known, some beneficial and harmful effects of treatments could have been identified earlier and might have prevented the conduct of the new trials. This would have led to the earlier uptake of effective health and social care interventions in practice, less exposure of trial participants to less effective treatments, and reduced waste resulting from unjustified research.’ ([17], p. 4)
Habre et al. (2014) [23]	To examine the effect of a 2000 systematic review of interventions preventing pain from propofol injection (the Picard review), which provided a clear research agenda, on the design of subsequent trials; to examine whether the designs of trials that cited the 2000 review differed from those that did not cite it; to establish whether the number of new trials published each year had decreased.	A comparison of the characteristics and design of trials published before and after the 2000 Picard review, which questioned the necessity to conduct further trials to identify another analgesic intervention to prevent pain from propofol injection. Parameters under comparison included blinding methods, the inclusion of children population, the use of the known most efficacious intervention as a comparator.	All RCTs investigating interventions to prevent pain from propofol injection in humans conducted and published after the Picard review.	136 new trial were conducted after the systematic review had questioned the necessity to conduct new studies. Only 36.0% of new trials could be considered to be clinically relevant as they used the most efficacious intervention as comparator or included a paediatric population as recommended by the review.	The impact of the Picard systematic review on the design of subsequent research was low. The number of trials published per year had not decreased; the most efficacious intervention was used only marginally.
Clayton et al. (2015) [32]	To summarise the current use of evidence synthesis in trial design and analysis; to capture opinions of trialists and methodologists on such use, and to understand potential barriers.	A survey collecting the views and experiences on the use of evidence synthesis in trial design and analysis.	638 participants of the International Clinical Trials Methodology Conference.	The response rate was only 17%. Respondents acknowledge that they had not been ‘using evidence syntheses as often as they felt they should’ [32], p. 1]. 42 out of 84 relevant respondents confirmed the use of meta-analyses to inform whether a trial is needed, while 62 out of 84 stated that this was desirable. Notably, only 6% of relevant respondents had applied earlier relevant evidence to inform sample size calculations, while 22% showed support for this. The main perceived barrier to the greater utilization of evidence synthesis in trial design or analysis was ‘time constraints, followed by a belief that the new trial was the first in the area’ ([32], p. 6).	Further research and training on how to synthesise and incorporate results from earlier trials can help ‘ensure the best use of relevant external evidence in the design, conduct and analysis of clinical trials’ ([32], p. 10).
Tierney et al. (2015) [33]	To identify the impact of individual patient data (IPD) meta-analyses on subsequent research in terms of the selection of comparators and participants, sample size calculations, analysis and interpretation of subsequent trials, as well as the conduct and analysis of ongoing trials.	Potential examples of the impact of IPD meta-analyses on trials were identified at an international workshop, attended by individuals with experience in the conduct of IPD meta-analyses and knowledge of trials in their respective clinical areas. Relevant trial protocols, publications, and Web sites were examined to verify the impacts of the IPD meta-analyses.	52 examples of IPD meta-analyses thought to have had a direct impact on the design or conduct of subsequent trials.	After screening relevant trial protocols and publications, 28 instances where IPD meta-analyses had clearly impacted on trials were identified. They have influenced the selection of comparators and participants, sample size calculations, analysis and interpretation of subsequent trials, and the conduct and analysis of ongoing trials, sometimes in ways that would not possible with systematic reviews of aggregate data. Additional potential ways of how IPD meta-analyses could be used to influence trials were identified in the course of the analysis.	IPD meta-analysis ‘could be better used to inform the design, conduct, analysis, and interpretation of trials’ ([33], p. 1326).
Storz-Pfennig (2016) [18]	To identify and estimate the extent, to which potentially unnecessary clinical trials in major clinical areas might have been conducted.	A cumulative meta-analysis and trial sequential analysis of a sample of Cochrane collaboration systematic reviews were conducted to determine, at what point evidence was found sufficient to reach a reliable conclusion. Trials published thereafter were considered potentially unnecessary and, therefore, wasteful. Sensitivity analysis was conducted in order to identify whether the findings could be explained by a delayed perception of published findings when planning new trials.	13 comparisons in major medical fields including cardiovascular disease, depression, dementia, leukemia, lung cancer.	In eight out of 13 comparisons, meta-analysis detected potentially unnecessary research with the range between 12 and 89% of all participants in trials that might not have been needed. In three of these cases with high proportions (69–89%) of potentially unnecessary research, this finding was found unchanged upon the sensitivity analysis.	‘The reasonableness of claims to relevance of additional trials needs to be much more carefully evaluated in the future. Cumulative, information size bases analysis might be included in systematic reviews. Research policies to prevent unnecessary research from being done need to be developed.’ ([18], p. 62)
De Meulemeester et al. (2018) [34]	To test the hypothesis that the majority of a sample of recently published RCTs would not explicitly incorporate the scientific criterion of addressing a persisting uncertainty established through a systematic review.	Cross-sectional analysis of all RCTs published in the *New England Journal of Medicine* and the *Journal of the American Medical Association* in 2015. The identified articles and protocols were reviewed inter alia for: a clearly stated central hypothesis, indications of evidentiary uncertainty, a meta-analysis or systematic review supporting the hypothesis or study question.	208 RCT articles and 199 protocols met the inclusion criteria.	The majority of RCTs (56%) did not meet the criteria of having a clear hypothesis and demonstrating that an uncertainty around that hypothesis exists, being established through a systematic review.	RCTs not meeting the criteria of having a clear hypothesis and demonstrating that an uncertainty around that hypothesis exists being established through a systematic review can be scientifically and therefore ethically unjustified. Authors recommend to replace the criteria of “equipoise,” “clinical equipoise,” and “lack of consensus” with the requirement that RCTs have a clearly stated, meaningful hypothesis around which uncertainty has been established through a systematic review of the literature.
Blanco-Silvente et al. (2019) [35]	To examine the strength of the available evidence on efficacy, safety and acceptability of cholinesterase inhibitors and memantine for Alzheimer’s disease (AD); To determine the number of redundant trials following the authorisation of cholinesterase inhibitors (ChEI) and memantine used as current pharmacological treatments for Alzheimer’s disease.	A cumulative meta-analysis with a trial sequential analysis, whereby the primary outcomes were cognitive function assessed with ADAS-cog or SIB scales, discontinuation due to adverse events and discontinuation for any reason. The redundancy of post-authorisation clinical trials was studied by determining the novel aspects of each study on patient, intervention, comparator and trial outcome characteristics. Two criteria of trial futility - lenient and strict – were used.	A total of 63 randomised clinical trials (RCTs) (16,576 patients) including placebo-controlled, double-blind, parallel-design RCTs with a minimum duration of 12 weeks that had investigated the effects of donepezil, galantamine, rivastigmine or memantine in monotherapy or in combination with a ChEI at the doses approved by the Food and Drug Administration or the European Medicine Agency in patients with Alzheimer’s disease.	It was conclusive that neither ChEI nor memantine achieved clinically significant improvement in cognitive function. In relation to safety, there was sufficient evidence to conclude that donepezil caused a clinically relevant increase on dropouts due to adverse events whereas the evidence was inconclusive for the remaining interventions. Regarding acceptability, it was conclusive that no ChEI improved treatment discontinuation while it was uncertain for memantine. The proportion of redundant trials was 5.6% with the lenient criteria and 42.6% with the strict criteria.	The evidence showed conclusively that neither ChEI nor memantine achieve clinically significant symptomatic improvement in Alzheimer’s disease, and that the acceptability of ChEI is unsatisfactory. Although evidence on the safety of pharmacological interventions for AD and acceptability of memantine is inconclusive, no further RCTs are needed as their efficacy is not clinically relevant. Redundant trials were identified but their number depends on the criteria of futility used.
Walters et al. (2020) [36]	To determine to what extent systematic reviews were cited as justification for conducting phase III trials published in high impact journals.	The analysis of all phase III RCTs published between 1 January 2016 and 31 August 2018 in *New England Journal of Medicine*, *Lancet*, and *JAMA*, in particular with regard to the references to systematic reviews as the justification for conducting the RCT in the introduction, methods, and discussion/conclusion. The strength of justification was classified as follows: (1) authors explicitly stated that a SR had established the need for the trial; (2) authors discussed the SR in a way that could be inferred that the SR provided the necessary justification; and (3) authors made no mention of using a SR as the basis for conducting the trial.	665 RCTs were retrieved, of which 637 were included that cited in total 728 systematic reviews.	Less than 7% of the analysed RCTs published in three high impact general medicine journals cited explicitly a systematic review as the basis for undertaking the trial.	Trialists should be required to present relevant systematic reviews to ethics committees demonstrating that the existing evidence for the research question is insufficient. Elimination of research waste is both scientific and ethical responsibility.

While it appears straightforward that a new trial should be initiated only if it is ‘necessary to address relevant uncertainty about the effects of one or more forms of health care’ ([37], p. 1391), evidence suggests that this fundamental principle of scientific research has often been neglected, and that trials continue being conducted – overall involving a large number of patients – long after the beneficial effect of a treatment has been established (see Table 1). Remarkably, in some cases, studies were ‘claimed to be the first trial even when many trials preceded them’ ([24], p. 54).

The cause of the problem is often attributed to the insufficient consideration of findings of earlier research and, especially, systematic reviews. Commentators have long argued that clinical trials ‘should begin and end with systematic reviews of relevant evidence’ [38], and that ‘research funders and regulators should demand that proposals for additional primary research are justified by systematic reviews showing what is already known, and increase funding for the required syntheses of existing evidence’ ([9], p. 156). Yet, evidence suggests that only a small fraction of RCTs explicitly reference systematic reviews as the justification for undertaking a new trial [36]. Even though the non-citation of relevant systematic reviews, in and of itself, does not render a trial redundant, it does raise a question as to what knowledge base supports the research hypothesis.

Needless to say, redundant trials are, first and foremost, unethical as they unjustifiably expose patients to health risks ([26], p. 88). They also violate the scientific principle that ‘the progress depends on new research being carried out and interpreted in the context of systematic reviews of all other relevant and reliable evidence’ ([39], para. 6.C.20.2). The opportunity cost of such studies corresponds to knowledge gaps that remain unaddressed [40–42], as well as inefficiencies in the allocation of resources due to missed opportunities to make the design of subsequent trials more informed and targeted ([29], p. 984).

Even though redundancy can hardly be quantified, several studies summarised in Table 1 attempted to measure the scope of the problem.

#### Earlier proposals and initiatives

The issue of the under-use of systematic reviews in the planning and design of new trials and the need for the greater scrutiny of trial applications in this regard have been discussed in the medical community, at least, from the late 1980s. Highlighting the need for the thorough examination of the existing evidence when new trials are planned and designed, Carpenter refers to the example reported in 1989, where studies had continued to investigate the effect of prophylactic antibiotics on the risk of infection after caesarean section for nearly two decades after the beneficial effect of antibiotics had been established ([43], p. 222). In 1993, Herxheimer put forward a proposal that a clinical trial ‘should be accompanied by a thorough review of all previous trials that have examined the same and closely related questions’ ([44], p. 211). In 1996, Savulescu, Chalmers and Blunt alleged that RECs ‘are behaving unethically by endorsing new research which is unnecessary’ ([37], p. 1390). They insisted that proposals for new trials should be supported by ‘scientifically defensible reviews of the results of relevant existing research’ ([ibid], p. 1391). Commentators have been sceptical, however, as to whether this requirement can be effectively enforced. For instance, Robinson and Goodman observe that there are ‘no barriers to funding, conducting, or publishing an RCT *without proof* that the prior literature had been adequately searched and evaluated’ ([24], p. 54, emphasis added), and that institutional review boards have ‘neither the capacity nor the charge to second-guess a researcher’s claim that a new RCT is needed’ (ibid).

An early attempt to institutionalise the requirement to submit systematic reviews can be traced to 1997 when the Danish national REC reportedly adopted a guidance requiring ‘applicants for ethical approval to show that they have carried out a full systematic review of the relevant scientific literature before the study will be approved’ ([45], p. 1189). According to Goldbeck-Wood, the initiative was driven by then Chairman of the Danish national REC Povl Riis, who believed that ‘me too studies’ are ‘unethical, because they randomise patients to receive a placebo intervention or drug, when an active drug is already known to exist [and] also waste valuable research funds without adding any new information, and drain the precious resource of appropriate control groups’ [ibid]. The provision, however, has not survived to date, and the current Danish Act on Research Ethics Review of Health Research Projects does not explicitly mention systematic reviews but only lists among the conditions for the authorisation that a proposed research project ‘should lead to new knowledge or investigate existing knowledge, which justifies the implementation of the research project’ ([46], section 18(1)(3)).

In 2014, the UK Health Research Authority developed a guidance ‘Specific questions that need answering when considering the design of clinical trials’ [47]. The authors of the guidance emphasise that the trial design ‘should be underpinned by a systematic review of the existing evidence, which should be reported in the protocol’ ([47], p. 2).

As an example of an editorial policy, *The Lancet* introduced a requirement that, as of 1 January 2015, authors submitting research papers to any journal within *The Lancet* group must include a section ‘Research in Context’, in which they have to describe all evidence, as well as its sources, that was taken into consideration prior to undertaking the study and indicate what value their findings can add to the existing evidence. Furthermore, the explanatory paper [48] accompanying the SPIRIT (Standard Protocol Items: Recommendations for Interventional Trials) Statement [49] recommends that relevant evidence such as systematic reviews should be cited in the protocol to support a proposed trial.

Given that publication and reporting guidance documents are not legally binding, the only instrument that can perform the ‘gatekeeping’ function is the regulatory authorisation of trials, in particular, requirements regarding the quality and the assessment of trial applications. While current practices of examining trial applications vary among the EU Member States, a consistent and harmonised approach is crucial for ensuring research quality throughout the Union. In what follows, we examine whether the EU Clinical Trials Regulation [25] can provide a relevant legal basis to support the above-mentioned initiatives in a unified way.

### Specific provisions that can be leveraged against redundant trials

The revision of the EU Clinical Trials Directive [50], mainly, pursued the following objectives: first, to modernise the regulatory framework for the submission, assessment, and regulatory follow-up of applications for clinical trials; second, to adapt regulatory requirements to practical considerations and needs; third, to address the global dimension of clinical trials when ensuring compliance with good clinical practice (GCP) ([51], p. 29–31). Towards those ends, the EU Clinical Trials Regulation introduced a streamlined application procedure through the EU portal, the concept of ‘low-intervention’ studies, and the EU database for clinical trial data.

Even though the revision of the EU Clinical Trials Directive did not tackle the issue of ‘wasteful research’, some provisions under the adopted EU Clinical Trials Regulation – if applied and enforced appropriately – can be instrumental in this regard. In particular, new requirements for the publication of trial data and the establishment of the new EU database under Article 81 can reduce waste resulting from the lack of transparency. As far as the issue of research redundancy is concerned, relevant provisions can be found among the requirements for the trial authorisation, in particular, those related to the methodology of a proposed study summarised in Table 2.

**Table 2:** An overview of the provisions under the EU Clinical Trials Regulation related to the justification of a clinical trial in light of the prior research

Provisions under the EU Clinical Trials Regulation	Text of the regulatory provisions (emphasis added)	Aspects that give a leeway for interpretation and the potential to reduce redundancy
Article 6 (1)(b)(i) second indent	An **application** for a clinical trial shall be assessed with regard to the anticipated therapeutic benefits and taking into account factors such as the *relevance* of the clinical trial and *the current state of scientific knowledge*.	•The notions of ‘trial relevance’ and ‘the current state of scientific knowledge’ are broad and can be subject to diverging interpretations. •‘Relevance’ of a trial might or might not be interpreted as the *need* to conduct a new trial in view of the existing evidence.
Article 25 (1)(a)	The **application dossier** for the authorisation of a clinical trial shall contain all required documentation and information necessary for its validation and assessment including the *scientific context*.	•The notion of ‘scientific context’ is subject to interpretation, especially as far as the scope is concerned. •Systematic reviews of prior studies and critical analysis of the existing evidence are not explicitly required.
Article 2 (23)	The **investigator’s brochure** (where applicable) that has to be submitted within the application for the trial authorisation is defined as a compilation of the clinical and non-clinical data on the investigational medicinal product or products which are *relevant* to the study of the product.	•The requirement concerns data on the experience not only with the investigational medicinal product but also *other products* which are relevant for the study.
Annex I(E)(25)	Investigator’s brochure has to be prepared in accordance with the state of scientific knowledge and international guidance.	•The criterion ‘in accordance with the state of scientific knowledge’ is of general nature. For instance, a trial can be designed in accordance with the principles and rules of medical statistics – yet, the research question that it intends to address may lack clinical relevance*.*
Annex I (E)(27)	The information in the investigator’s brochure shall be presented in a concise, simple, objective, balanced and non-promotional form that enables a clinician or investigator to understand it and make an unbiased risk-benefit assessment of the *appropriateness* of the proposed clinical trial. It shall be prepared from *all* available information and evidence that supports the *rationale* for the proposed clinical trial and the safe use of the investigational medicinal product in the clinical trial and be presented in the form of summaries.	•The notion of ‘appropriateness’ in conjunction with the ‘trial rationale’ can be interpreted as the requirement to show that the study intends to resolve a persisting clinical uncertainty that can justify the risks and costs involved. •The requirement to base the rationale on ‘all available information and evidence’ presupposes extensive search on the part of investigators.
Annex I (G)(46),(47)	The **investigational medicinal product dossier** shall provide *summaries* of all available data from previous clinical trials and human experience with the investigational medicinal products.	•The requirement concerns data on the experience only *with* the investigational medicinal product.
Annex I (D)(17)(c)	The **trial protocol** shall include a *summary of findings* from non-clinical studies that potentially have clinical significance and from *other clinical trials* that are *relevant* to the clinical trial.	•Notably, the scope of earlier evidence, which has to be taken into consideration, extends to other trials that can be relevant for the proposed study. •Only references and summaries of findings from previous studies are required to be submitted. Neither systematic reviews, nor critical assessment of earlier studies, nor the explanation of how they informed the design of a proposed trial are explicitly required. •The notion of ‘relevant’ literature and data that form the scientific background can be interpreted expansively.
Annex I (D)(17)(d)	The trial protocol shall include a summary of the known and potential risks and benefits including an evaluation of the anticipated benefits and risks to allow assessment in accordance with Article 6	
Annex I (D)(17)(i)	The trial protocol shall include references to literature and data that are *relevant* to the clinical trial, and that provide background for the clinical trial.	

Several limitations of the identified provisions can be pointed out. Most importantly, neither systematic reviews, nor critical assessment of earlier studies are explicitly required. The scientific background has to be provided in the form of references to relevant literature and studies. A mere referencing of literature is, however, insufficient as it does not involve the analysis and synthesis of the evidence. Neither is it clear whether and how the referenced literature actually informed the design of a proposed trial. While ‘the scientific context’ and ‘the current state of scientific knowledge’ have to be presented, the question arises as to how they ought to be assessed, especially in terms of the quality and completeness. The scope of the regulatory mandate of institutions in charge of the trial authorisation appears to be ambiguous in this regard. If approached formalistically, a critical analysis of whether the proposed study, in the way it has been designed, is necessary and justified might be missing.

Next, only data on the investigational medicinal product is explicitly required to be submitted within an investigational medicinal product dossier. If, for instance, a new beta blocker was to be tested in patients with myocardial infarction, a systematic review of evidence on the available beta blockers in patients with myocardial infarction – and, most importantly, whether a substantially efficacious product or method of treatment already exists – might be neglected. Even though the EU Clinical Trials Regulation promulgates the overarching principle of ensuring data reliability and robustness ([25], Articles 3 and 6(1)(b)(i)), this principle corresponds to the *internal* validity of a trial, which means that a trial is designed in a way that it answers the research question in a reliable way ([52], p. 8:2). However, whether a study asks a clinically relevant question to begin with, lies outside the concept of the trial internal validity.

At the same time, it is important to highlight that several provisions broaden the scope of the prior research that ought to be taken into account, such as the requirements to submit data on *other relevant products* within the investigator’s brochure and findings from *other relevant studies* within the trial protocol. The beneficial aspect of the broad language of the provisions – in particular, the requirements concerning the relevant ‘scientific context’, ‘relevant literature and data’ and ‘trial appropriateness’ – can be seen in that, first, such language enlarges the scope of the scientific background beyond prior evidence *on* the investigational product that needs to be taken into consideration when a new trial is planned, designed and assessed; second, it leaves a leeway for interpretation. Accordingly, the effectiveness of the EU Clinical Trials Regulation in addressing the problem of redundancy critically depends on how these requirements are applied by institutions involved in the trial authorisation.

### The proposed interpretation and justification

In view of the above-outlined considerations, we propose that the requirements for trial applications and their assessment – in particular, under Article 6(1)(b)(i) second indent and Article 25(1)(a) of the EU Clinical Trials Regulation – shall be interpreted and applied as:

- a duty on applicants for the trial authorisation to justify the need to conduct a new trial by demonstrating that it addresses an *outstanding clinical uncertainty* based on the critical assessment of the accessible relevant evidence from earlier research, including in the form of systematic reviews, and
- a duty on the institutions in charge of the authorisation of clinical trials – typically, NCAs and RECs – to require and critically examine such justification.

In what follows, we explain the main rationales supporting the proposal.

#### Preventing unnecessary trials as a matter of protection of the well-being of trial participants

The proposal is supported by the teleological method of interpretation that construes the meaning of legal provisions in light of the underlying policy objectives and principles. Ethical integrity and scientific quality of clinical trials are the core values that lie at the heart of the EU Clinical Trials Regulation. The protection of the rights, safety, dignity and well-being of subjects, as well as ensuring reliability and robustness of clinical trial data constitute the main objective and the general principle of conducting interventional studies ([25], Recital 85, Article 3). These values correspond to the universal ethical principles proclaimed under the 1964 Declaration of Helsinki [53] and the overarching notion of good clinical practice. The latter is defined under the 1996 Guideline of the International Council for Harmonisation of Technical Requirements for Pharmaceuticals for Human Use (ICH) as ‘[a] standard for the design, conduct, performance, monitoring, auditing, recording, analyses, and reporting of clinical trials that provides assurance that the data and reported results are credible and accurate, and that the rights, integrity, and confidentiality of trial subjects are protected’ ([54], para. 1.24). In fact, the wording of the objectives and principles under the EU Clinical Trials Regulation ([25], Recital 85, Article 3) is a slight paraphrase of the definition of GCP under the ICH Guideline.

The fundamental ethical principle of medical research in humans posits that health risks borne by study subjects can only be justified by knowledge that *could not be otherwise* obtained ([26], p. 1). Accordingly, it appears straightforward that preventing redundant trials shall be viewed as a matter of protection of the rights, safety and well-being of trial participants and, thus, come under the purview of RECs.

#### The necessity of a trial as a matter of the ethical assessment

Ambiguity can arise as to what institutions shall examine the necessity of a trial *vis-à-vis* the prior knowledge. Article 4 of the EU Clinical Trials Regulation stipulates that a trial application shall be subject to both ethical and scientific review. The division of tasks and responsibilities between RECs and NCAs is, however, not harmonised in this regard. Notably, the participation of RECs in the evaluation of aspects related to the trial methodology, including the trial relevance and scientific context, is optional. Article 4 of the EU Clinical Trials Regulation reads:

The ethical review shall be performed by an ethics committee in accordance with the law of the Member State concerned. The review by the ethics committee *may* encompass aspects addressed in Part I of the assessment report for the authorisation of a clinical trial as referred to in Article 6 and in Part II of that assessment report as referred to in Article 7 as appropriate for each Member State concerned (emphasis added).

Besides, recital 18 of the EU Clinical Trials Regulation states that the Member States have full discretion ‘to determine the appropriate body or bodies to be involved in the assessment of the application to conduct a clinical trial and to organise the involvement of ethics committees’.

The current situation varies among the EU Member States in this regard: In some countries, such as Greece [55], RECs are in charge of only part II of the assessment report (ie ethical aspects, such as informed consent and compensation issues, according to Article 7 of the EU Clinical Trials Regulation), while part I (the scientific assessment pursuant to Article 6 of the EU Clinical Trials Regulation) is evaluated by other competent bodies. In other countries, such as Germany, the RECs make the overall assessment including the scientific quality, legitimacy and ethical justifiability [56, 57].

It is important to emphasise that the distinction between the ethical and scientific review is notional only. The two aspects cannot be separated because scientifically unsound research involving humans is unethical as it exposes trial participants to unjustified health risk ‘for no purpose’ ([26], p. 88). As stressed by the Guidelines of the Council for International Organizations of Medical Sciences (CIOMS), RECs ‘must [ …] recognize that the scientific validity of the proposed research is essential for its ethical acceptability’ and, therefore, ‘must either carry out a proper scientific review, verify that a competent expert body has determined the research to be scientifically sound, or consult with competent experts to ensure that the research design and methods are appropriate’ ([26], p. 88). The UNESCO Universal Declaration on Bioethics and Human Rights states: ‘Independent, multidisciplinary and pluralist ethics committees should be established, promoted and supported at the appropriate level in order to [*inter alia*] assess the relevant ethical, legal, *scientific* and social issues related to research projects involving human beings’ ([58], article 19(a), emphasis added).

As a legally non-binding but norm-setting instrument laying down ethical principles of conducting medical research in humans, the 2012 Guide for Research Ethics Committee Members of the Council of Europe [39] makes an explicit reference to systematic reviews in relation to the trial scientific quality and justification as the prerequisite for the trial authorisation. In particular, it states that RECs ‘must be satisfied about the scientific quality of the research proposal’ ([39], para. 5.A.1.1), that they ‘should pay particular attention to the scientific justification for the proposed research [in order to] help prevent inappropriate research’, and that systematic reviews of research results, in animals as well as human beings are ‘especially important’ in that regard ([39], 6.C.1). Furthermore, the Guide mentions that the ‘aim of and justification for the research based on the most up-to-date review of scientific evidence’ ([39], para. 6.C) should be stated in the description of the study, which is provided to and examined by RECs.

In sum, there is no doubt that RECs have all reasons and discretion to evaluate the necessity of a newly proposed trial – and, towards this goal, require systematic reviews – as a part of the ethical assessment of trial applications.

#### A mere reference of a systematic review is not sufficient

Systematic reviews can aid in designing, conducting, and analysing clinical trials in numerous ways, including by informing the choice of comparator, sample size calculation, eligibility criteria, selection and definition of the trial outcomes [31, 59–62]. Thus, apart from referencing relevant systematic reviews, it is important that a trial application should demonstrate how they informed the design of a proposed trial. The study conducted by Habre et al. [23] illustrates this point. While a 2000 systematic review already questioned the necessity of performing new trials to identify another analgesic intervention to prevent pain from propofol injection, 136 trials were subsequently conducted. Remarkably, the authors were unable to identify significant differences between the designs of the trials citing and not citing the systematic review at issue ([23], p. 5). Thus, without the explicit explanation, it cannot be taken for granted that the referenced literature, in fact, informed the design of a new study.

### Where to draw the line?

The proposal to apply the requirement of justifying new trials against the background of relevant systematic reviews more stringently can be viewed as an ‘obvious remedy’ ([24], p. 54). Several critical aspects require, nevertheless, further methodological reflection.

#### Redundancy vs. genuine uncertainty

In some cases, redundancy can be evident, especially where robust efficacy of a treatment has been clearly demonstrated, such as in the astounding cases described by Habre et al. [23], where 136 trials involving 19,778 patients were conducted after the systematic review had questioned the necessity of conducting new studies; by Fergusson et al. [21] where 52 RCTs were conducted to address the question of efficacy that had been established definitively by prior research; or Ker et al. [30] where trials were conducted 10 years after reliable evidence confirmed the treatment effect at issue. In some situations, an outcome of interest can be derived by way of scientific inference, whereby biological insights into the mechanism of action of a treatment and knowledge of relevant factors can inform the decision-making ([63], p. 6). The question of whether scientific inference can be justified has to be assessed on the case-by-case basis.

However, in other cases, there can be a fine line between the instances where a research question can be answered conclusively on the basis of the accessible evidence, and where clinical uncertainty may still persist. Even if efficacy of a treatment has been demonstrated, subsequent studies can be justified if they seek to examine uncertain aspects of a treatment, optimise the dosage regimen, or find a more favourable benefit-risk ratio. Such differences vis-à-vis prior research should be reflected in the objectives of a newly proposed RCT and the way it was designed, especially as far as the definition of the outcome of interest and endpoints is concerned. For instance, when commenting on the study by Fergusson et al., Augoustides and Fleisher suggested that intention to find a more cost-effective dosage regimen, or a better benefit-risk balance, is a possible reason for redundancy in trials ([64], p. 231–2). That, however, was not the case: the follow-on trials examined by Fergusson et al. displayed homogeneity in terms of their objectives and outcome measures ([21], p. 224).

#### Replicability vs. redundancy

While the recommendation that replication ‘to check the validity of previous research is justified, but unnecessary duplication is unethical’ ([65], p. 2) is sensible, in many cases, it might be difficult to draw the line between redundancy and the need to ensure generalizability. The latter means that the study results can be applied in the contexts or populations other than the original one. Generalizability relates to replicability of medical knowledge, ie the ability to replicate the data from an earlier study by following the same procedures ([66], p. 4). Both, replicability and generalizability are difficult to achieve in RCTs due to the biological variability of study subjects and diseases.

Notably, the study by Ker and Roberts [63] found that concerns about the generalizability of the results of earlier studies – including due to the change in patient characteristics – are often indicated as the main motivation for new trials. They assume that the awareness of systematic reviews confirming a reliable demonstration of a treatment effect can, in fact, stimulate an increase rather than decrease in trial activity, as investigators would be motivated to confirm the treatment effect in a different population. In such cases, potential redundancy can only be detected through a thorough analysis as to whether the generalizability and replicability of findings from earlier relevant studies can be called into question. Such assessment, in turn, crucially depends on the accessibility of the trial protocols from prior studies. Access to non-summary level of clinical trial data has been, and still is, challenging in many jurisdictions. At the same time, policy measures such as the establishment of the EU Clinical Trial database, new transparency requirements providing for the publication of clinical study reports ([25], Article 81), the policy on access to trial data of the European Medicines Agency [67], as well as publication policies of medical journals [68] can, to a significant extent, alleviate the problem and enable secondary data analyses.

#### Reliability of data from earlier studies and systematic reviews – a vicious cycle?

Another critical factor is the reliability of data from earlier studies and the quality of systematic reviews. Commentators point out the issue of exponential production of redundant, potentially conflicting and misleading systematic reviews and meta-analyses [69, 70]. Some argued that many systematic reviews ‘fail to provide a complete and up-to-date synthesis of evidence’, and that ‘failure to rigorously synthesize the totality of relevant evidence may have a detrimental effect on treatment decisions and future research planning’ ([71], p. 2]; [72, 73]). Low quality of meta-analyses is viewed to be a more significant cause of redundant research than the failure to appraise the existing evidence ([63], p. 1).

In light of these allegations, one may question whether investigators of newly proposed trials might be better-off by *not* relying on the conclusions drawn from the synthesis of the reported data. As a safeguard, which could alleviate such concerns, at least to some extent, systematic reviews referenced in trial applications should demonstrate the adherence to the recognised quality standards and methodological guidance, such as guidelines developed by the Cochrane Collaboration [74], as well as the established publication standards, guidelines, and principles [75, 76]. For instance, the study by Sun et al. [77] showed that, since the publication of the PRISMA Statement, the quality of reporting of systematic reviews and meta-analyses in the area of nursing interventions in patients with Alzheimer’s disease has improved. Besides, various analytical methods such as cumulative network meta-analysis [78–80] and analytical tools [81] can assist trialists in identifying relevant prior studies and managing exponentially growing trial data [82] and, ultimately, ‘prevent experimentation with an unnecessarily large number of participants’ ([79], p. 1).

#### The ‘best-in-class’ strategy vs. redundancy

To a large extent, follow-on trials are driven by the so-called ‘best-in-class ’[83, 84] competitive strategy of drug companies directed at the development of drug improvements. The ‘best-in-class’ competitive strategy implies that pharmaceutical companies aim at the improvement of drugs with a particularly advantageous economic profile, while the ‘first-in-class’ strategy pursues the development of ‘breakthrough’ drugs ([83], p. 12). Competition by drug improvements includes the development of new formulations, modes of administration, combinations of active ingredients with known therapeutic activity ([84], p. 49). The critical question is: At what point should research and development efforts addressing a particular condition cease and be diverted to unresolved clinical uncertainties, especially if a substantially efficacious treatment has already been identified among the alternatives?

The need for follow-on RCTs can only be determined on the case-by-case basis. While, in some situations, follow-on drugs might feature higher efficacy, reduced side effects, or a more convenient regimen ([85], p. 34–35), in other cases they represent insignificant modifications of the existing medicines that can be so minor and the clinical need for such modifications can be so small that the clinical benefit would not outweigh the costs of conducting trials.

In any event, the need for a new RCT should be considered carefully, as some clinical uncertainties under certain conditions can be adequately investigated without randomisation, eg where there is a good understanding of the mode of action, such as with ß-blockers, ACEI, or statins. There is a long-standing discussion in the medical research community concerning the conditions, under which a randomized trial is definitely needed [86, 87]. At the same time, evidence shows that regulatory approval of pharmaceuticals without randomized controlled studies is nowadays common by agencies such as the European Medicines Agency and the U.S. Food and Drug Administration [88].

In situations, where a new trial is conducted in the presence of an established treatment, it would be logical to expect that an investigational product shall be compared with that reference treatment, whenever ethically acceptable. When a randomised trial is planned, it has to be thoroughly examined whether access to the best effective treatment is limited (eg by a placebo control) beyond what is acceptable by the current ethical standards established under the Declaration of Helsinki [53]. Studies report disturbing evidence that randomised placebo-controlled trials continue to be the dominant study design for assessing pharmacological interventions [89]. Apart from obvious ethical concerns, studies where the use of placebo is unjustified can represent a significant source of waste as they do not generate knowledge regarding comparative benefits and risks of medical interventions [90].

It is important to emphasise that the use of placebo can be justified only where there is a genuine uncertainty as to whether one treatment is superior to placebo (the ethical principle of clinical equipoise) ([91], p. 141). The CIOMS International Ethical Guidelines for Health-related Research Involving Humans state that, as a general rule, study participants in the control group of a trial *must* receive an established effective intervention, where an established effective intervention exists for the condition under investigation ([26], p. 9, 15, 17). According to the ICH Guideline, for serious diseases, if a therapeutic treatment which has been proved to be efficacious by superiority trials exists, a placebo-controlled trial can be deemed unethical and ‘the scientifically sound use of an active treatment as a control should be considered’ ([92], p. 14). Accordingly, there is no doubt that the choice of control should constitute a part of the ethical assessment of trial applications.

#### Is it feasible at all?

When responding to the article by Savulescu, Chalmers and Blunt [37] calling on RECs to consider more critically the need for new studies, a representative of a REC contended that ‘it is unreasonable and unrealistic’ to expect the quality of medical research to be improved ‘entirely through the mechanism of review by local research ethics committees’ ([93], p. 676). This remark is accurate in that many RECs, as well as NCAs, lack human resources in the fields of research methodology and biostatistics. The Report of the European Commission [94] shows that, in many Member States, the number of quality and clinical assessors involved in the clinical trial assessment in the NCAs is extremely limited ([94], p. 22–23). Notwithstanding whether these individuals might be represented by physicians, pharmacologists, toxicologists, pharmacists or, in some rare case, biostatisticians, the workforce is highly disproportionate to the workload ([94], p. 28–29). In this view, it might be simply unfeasible for RECs to conduct an in-depth evaluation of the quality, relevance, and completeness of the submitted systematic reviews, or other background literature.

Thus, in reality, RECs ‘seem to stand alone, with limited resources and sometimes not enough scientific credit or knowledge to identify, and stop, the performance of irrelevant research’ ([23], p. 4). While such situation is regrettable, the point is that it should be viewed and remedied as the problem of lacking human resources, and not as the absence of the relevant legal basis for requiring stronger justification of new trials in light of prior research.

## Conclusions

In answering the question posed by the title, we have shown that the EU Clinical Trials Regulation clearly has a potential to effectively reduce redundant RCTs. The extent to which it can do so depends on how the requirements for the trial authorisation are interpreted and applied by the institutions concerned. The recommendations proposed in this article for exercising more stringency as to the justification of new trials in light of prior research can provide further guidance and be undoubtedly instrumental in this regard. The proposal is supported by the fundamental objectives and underlying principles of conducting research in humans that are promoted by the EU Clinical Trials Regulation – namely, the protection of the well-being of trial participants and ensuring data reliability and robustness –, as well as the overarching concept of good clinical practice. To the extent, to which these principles are applied by investigators, sponsors, RECs and other institutions involved in the authorisation and monitoring of clinical trials, the analysed regulatory provisions can be effective in tackling the problem of research redundancy.

The review of the EU Clinical Trials Regulation will not take place earlier than 2024 and is expected to address the regulatory impact on scientific and technological progress and the competitiveness of European clinical research ([25], Article 97). In view of the foregoing, we argue that it is research quality that shall be viewed as the principal component of competitiveness, and that the revision of the EU Clinical Trials Regulation should strengthen the methodological aspects of trial planning and design addressed by the present analysis. Until then, institutions responsible for the trial authorisation should rely on the regulatory provisions regarding the preparation and assessment of trial applications, identified and analysed in this article, as the legal basis to examine more stringently the necessity of new studies and their methodological quality.

## Data Availability

Not applicable.

## Abbreviations

CIOMS: The Council for International Organizations of Medical Sciences
EU: The European Union
GCP: Good clinical practice
ICH: The International Council for Harmonisation of Technical Requirements for Pharmaceuticals for Human Use
IPD: Individual patient data
NCA: National competent authority
RCT: Randomised clinical trial
REC: Research ethics committee
SR: systematic review
UNESCO: The United Nations Educational, Scientific and Cultural Organization
